# Copolymer Coatings for DNA Biosensors: Effect of Charges and Immobilization Chemistries on Yield, Strength and Kinetics of Hybridization

**DOI:** 10.3390/polym13223897

**Published:** 2021-11-11

**Authors:** Luka Vanjur, Thomas Carzaniga, Luca Casiraghi, Giuliano Zanchetta, Francesco Damin, Laura Sola, Marcella Chiari, Marco Buscaglia

**Affiliations:** 1 Dipartimento di Biotecnologie Mediche e Medicina Traslazionale, Università degli Studi di Milano, 20054 Segrate, Italy; luka.vanjur@unimi.it (L.V.); thomas.carzaniga@unimi.it (T.C.); luca.casiraghi@unimi.it (L.C.); giuliano.zanchetta@unimi.it (G.Z.); 2 Istituto di Scienze e Tecnologie Chimiche, Consiglio Nazionale delle Ricerche (CNR-SCITEC), 20131 Milano, Italy; francesco.damin@scitec.cnr.it (F.D.); laura.sola@scitec.cnr.it (L.S.); marcella.chiari@scitec.cnr.it (M.C.)

**Keywords:** DNA hybridization, label-free detection, DNA hybridization kinetics, copolymers

## Abstract

The physical–chemical properties of the surface of DNA microarrays and biosensors play a fundamental role in their performance, affecting the signal’s amplitude and the strength and kinetics of binding. We studied how the interaction parameters vary for hybridization of complementary 23-*mer* DNA, when the probe strands are immobilized on different copolymers, which coat the surface of an optical, label-free biosensor. Copolymers of *N*, *N*-dimethylacrylamide bringing either a different type or density of sites for covalent immobilization of DNA probes, or different backbone charges, were used to functionalize the surface of a Reflective Phantom Interface multispot biosensor made of a glass prism with a silicon dioxide antireflective layer. By analyzing the kinetic hybridization curves at different probe surface densities and target concentrations in solution, we found that all the tested coatings displayed a common association kinetics of about 9 × 10^4^ M^−1^·s^−1^ at small probe density, decreasing by one order of magnitude close to the surface saturation of probes. In contrast, both the yield of hybridization and the dissociation kinetics, and hence the equilibrium constant, depend on the type of copolymer coating. Nearly doubled signal amplitudes, although equilibrium dissociation constant was as large as 4 nM, were obtained by immobilizing the probe via click chemistry, whereas amine-based immobilization combined with passivation with diamine carrying positive charges granted much slower dissociation kinetics, yielding an equilibrium dissociation constant as low as 0.5 nM. These results offer quantitative criteria for an optimal selection of surface copolymer coatings, depending on the application.

## 1. Introduction

Capturing DNA or RNA strands with specific sequences by surface-immobilized complementary probe strands is the basis of established DNA microarray technology [1] and many innovative DNA biosensors [2]. Fluorescence-based DNA microarrays can simultaneously provide profiling expression of thousands of genes, and are widely used in many biomedical applications [3]. More generally, the formation of a double strand from two complementary sequences, called hybridization, can be coupled to different mechanisms for signal transduction [4]. In label-free biosensors, the signal originates directly from the presence of the target strands through the change of some physical properties of the interface hosting the probes, such as mass [5], electrical conductivity [6], or refractive index [7]. In all these cases, the detection performance relies on the molecular recognition process between the two complementary strands. Many studies have shown that nucleic acid hybridization with surface-immobilized probe strands provides different features than the same process occurring between strands freely diffusing in solution [8,9]. Despite such an accumulation of evidence, a comprehensive understanding of the phenomena affecting surface hybridization is still missing, and quantitative comparison between different surface treatments for covalent immobilization of probes and passivation remains challenging to achieve.

Among other factors, the physical and chemical properties of the surface can strongly affect the robustness of detection [2,10,11]. Besides the influence of nonspecific binding due to the passivating capability of the surface treatment, the immobilization strategy and the local probe environment affect the density and orientation of the tethered DNA strands, which impact the sensitivity and specificity of the technique [12]. Additionally, the electrical properties of the sensing interface, including the accumulation of ionic species, have a direct effect on the strength and kinetics of surface hybridization [13].

Multifunctional coatings based on copolymers of *N*, *N*-dimethylacrylamide (copoly-DMA), forming a nanoscale film, have been widely employed to realize microarrays and multispot biosensors on different substrates [14,15,16,17]. This family of copolymers provides rapid and robust surface adhesion [18], covalent coupling of biomolecules and excellent antifouling capacity [19]. The precursor of this polymer family bears a functional group, *N*-Acryloyloxysuccinimide (NAS), that reacts with amines, and consequently, easily binds protein, peptide and amino-modified oligonucleotides [20]. Alternatively, the copolymer can be enriched by functional groups enabling click-chemistry reactions, such as azide/alkyne reactions, which improve stability and extend the coating’s shelf life [15,20]. Moreover, click-chemistry reactions provide the biorthogonal orientation of the immobilized probe, which has been demonstrated to positively affect the analytical outcome of microarrays, especially for serological applications [21].

In this work, we investigated the effect on hybridization parameters of different immobilization strategies and local environments of 23-*mer* DNA probes tethered to different variants of copoly-DMA. We measured hybridization with complementary strands in solution with a Reflective Phantom Interface (RPI), label-free, optical biosensor [7,13,22,23], which enabled us to extract surface densities of immobilized probes, kinetic rate constants, equilibrium constants and amount of target captured at saturation. The results confirmed a strong effect of probe surface density on the association kinetics for all copolymers. We obtained a quantitative comparison of different surface treatments by considering this dependence. Despite the similar composition of the polymer backbone, we observed significant effects on hybridization due to the density and type of probe immobilization sites and electrophilicity of the copolymer. Azide/alkyne immobilization provided the highest amounts of captured targets at saturation, but reduced affinities. In contrast, a large density of active ester-based immobilization sites showed the highest hybridization affinity but a slightly lower signal amplitude. Moreover, we observed that adding positive charges to the copolymer backbone slows down the dissociation kinetics. The results of this work are expected to guide the derivation of quantitative criteria for selecting optimal surface coatings, depending on the target parameter and the measuring conditions of the assay.

## 2. Materials and Methods

### 2.1. Copolymer Surface Coatings

**Figure 1:**
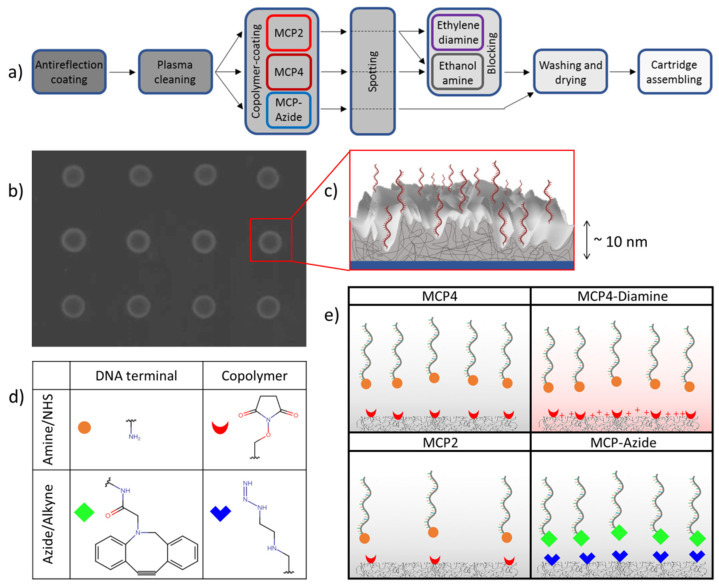
RPI sensor surface coated by DMA-based copolymers and spotted with DNA probes. (**a**) Process scheme for RPI biosensor fabrication. (**b**) RPI image of a glass prism spotted with single-strand DNA with surface density 0.4 ng/mm^2^. Spot diameter is about 150 μm and spot-to-spot distance is 400 μm. (**c**) Cartoon of the copolymer hydrogel on the sensor surface. Single strands of 23-*mer* DNA (red) are immobilized at the 5′ terminal on the surface (shaded silver grey) of a 3D copolymer layer (gray with lines) coating the SiO_2_ layer (blue). (**d**) Functional groups for covalent immobilization of DNA probe strands on copolymers. (**e**) Schematic of the four variants of DMA-based copolymers. Colored symbols represent the functional groups of panel (**d**).

RPI glass sensors were coated with different copolymers, providing covalent immobilization of DNA probe strands. Following the procedure described in [22], wedge-like glass chips (F2 optical glass, Schott, Mainz, Germany) with a 5° angle, with maximum thickness of 2 mm and a size of 8 mm × 12 mm, were coated with SiO_2_ to form an antireflection layer of about 79 nm, providing a minimum of reflectivity in the blue spectral region in conditions of normal incidence. As described in [20], before coating with copolymers, the chips were pretreated with oxygen plasma for 10 min: the oxygen pressure was set to 1.2 bar with a power of 29.6 W. Each copoly-DMA was dissolved in DI water to a final concentration of 2% *w/v* and then diluted 1:1 with a solution of ammonium sulphate 1.6 M. Each RPI chip was immersed into a copoly-DMA solution for 30 min, then rinsed with DI water, dried under a nitrogen stream, and finally cured under vacuum at 80 °C for 15 min. Then, the chips were spotted as described in Section 2.3. The fabrication process of RPI biosensors is summarized in Figure 1a.

The studied copolymers are all compounds made of dimethylacrylamide (DMA), 3-(trimethoxysilyl)propyl methacrylate (MAPS) and of a third monomer, either NAS or azide [20]. The copolymers differ in the amount or type of reactive sites for the DNA probes or on the net charge added in the final passivation step (Figure 1). The DMA:NAS:MAPS comonomer molar ratio is 89:10:1 for MCP4 and 97:2:1 for MCP2. Therefore, the fraction of amine-reactive sites of MCP4 is five-times larger than that of MCP2. In both cases, after the immobilization of the DNA probes, the reactive sites of the copolymer were passivated with ethanolamine [24]. In the case of MCP4-Diamine the passivation was obtained by using ethylenediamine, in order to provide the copolymer structure with a positive net charge in water [25]. A different copolymer variant, MCP-Azide, was obtained by substituting NAS residues with azide groups by postpolymerization modification reactions in the copolymer formulation [20], hence enabling click-chemistry immobilization of the DNA probes conjugated with DBCO. MCP4 and MCP2 copolymers were purchased from Lucidant Polymers (Sunnyvale, CA, USA). MCP4-Diamine and MCP-Azide were prepared in the laboratory according to common protocols [20], using buffers and reagents purchased from Sigma-Aldrich (St. Louis, MO, USA) and Milli-Q pure water.

### 2.2. DNA Strands and Reagents

We studied the hybridization of 23-*mer* single-strand DNA (ssDNA) in solution with complementary probe strands immobilized on the RPI biosensor surface. We selected this sequence length as a general model for a stable paring and without significant self-pairing. Similar DNA lengths are commonly exploited for PCR primers, or in structural DNA-nanotechnology applications [26]. Moreover, 23 bases represents the average length of micro-RNA (miRNA) biomarkers, a class of naturally occurring, small, noncoding RNA molecules, which are the target of several microarrays or biosensors [23,27]. The sequence of the surface-immobilized probe was: 5′-GCCCACCTATAAGGTAAAAGTGA-3′. The probe strand was modified at the C6 carbon of the 5′ terminal with amine or DBCO, in order to react with and covalently bond to NHS or azide on the copolymer surface coating, respectively. The target sequence added into the solution during the experiments was fully complementary to the probe. Both the probe and target strands were purchased from Integrated DNA Technologies (Leuven, Belgium) with high-quality Ultramer synthesis.

### 2.3. RPI Sensor Preparation

DNA probe strands were covalently immobilized on the surface of RPI sensing chips in spots with 150–200 μm diameter. Droplets of spotting buffer (Na_2_HPO_4_, pH 8.5, 150 mM and sucrose monolaurate 0.01% *w/v*) containing amine- or DBCO-terminated DNA probes at concentrations *C_P_* from 1 up to 30 μM were deposited on the chip surface by an automated, noncontact dispensing system (sciFLEXARRAYER S5; Scienion AG, Berlin, Germany). After overnight incubation, the chip surface was rinsed with blocking buffer (Tris-HCl, pH 8, 10 mM, NaCl 150 mM, ethanolamine 50 mM) and distilled water and then dried. For the samples with MCP4-Diamine coating, ethanolamine was replaced by ethylenediamine at the same concentration in the blocking buffer. The sensor cartridges were prepared by gluing the glass chips onto the inner wall of 1 cm plastic cuvettes. The cartridges were stored at 4 °C before use. The target DNA strands were suspended before use in measuring buffer (10 mM Tris-HCl, 0.02% NaN_3_, NaCl 150 mM, pH 8.0).

### 2.4. RPI Measurement and Analysis

The RPI measurements were performed using the apparatus and the analysis algorithm described in [22]. The sensor cartridges were filled with 1.3 mL of measuring buffer. The cartridges were kept at 23 °C during the measurement through a thermalized holder, and rapid mixing of the solution was provided by a magnetic stirring bar. Sample spikes of target ssDNA were performed by adding 50 μL of measuring buffer containing different amounts of target molecules to a final concentration in the cartridge from 0.5 nM up to ~300 nM.

Time sequences of RPI images of the spotted surface were analyzed by a custom MATLAB program (The MathWorks, Natick, MA, USA) to obtain the brightness of each spot as a function of time *t*, and converted into the total mass surface density of molecules σ(t). The conversion of the brightness of the RPI image pixels *u_s_*(*t*) into surface density is performed according to:

(1) $$\sigma \left(t\right)=\sigma^{*}\sqrt{\frac{u_{s}\left(t\right)}{u_{o}}-1}-\delta \sigma$$

where $\sigma^{*}$, $u_{0}\;$ and δσ are obtained as described in [22] from the physical parameters of the RPI sensor, the refractive index of the solution, and the density and refractive index of a compact layer of biomolecules on the surface. The mass surface density of the target molecules is obtained as $\sigma_{T}\left(t\right)=\sigma \left(t\right)-\sigma_{P}\left(t\right)$, where $\sigma_{P}\left(t\right)$ is the mass surface density of immobilized probe molecules measured before the addition of the target ssDNA in solution. The analysis of the hybridization curves was performed on $\sigma_{T}\left(t\right)$ traces obtained by averaging at least six spots with identical composition. The values for surface density of probe, $S_{P}$, and target molecules, $S_{T}$, were obtained by dividing $\sigma_{P}$ and $\sigma_{T}$ by the probe molecular mass, respectively. Hybridization kinetic curves were analyzed under the framework of Langmuir model [19]. Each binding response that followed the addition of target strands into the cuvette was fitted with the exponential growth function:

(2) $$\sigma_{T}\left(t\right)=\left(\sigma_{eq}\left(C_{T}\right)-\sigma_{T}\left(0\right)\right)\;\left(1-e^{-k_{obs}t}\right)+\sigma_{T}\left(0\right)$$

where:

(3) $$\sigma_{eq}\left(C_{T}\right)=\frac{\sigma_{\infty }}{1+\frac{K_{d}}{C_{T}}}$$

is the equilibrium plateau value, which depends on the dissociation equilibrium constant $K_{d}=k_{off}/k_{on}$ of probe–target hybridization and on the mass surface density at saturation $\sigma_{\infty }$, and

(4) $$k_{obs}\left(C_{T}\right)=k_{on}C_{T}+k_{off}$$

is the observed hybridization rate. The value of surface density of target, $S_{T}\left(t\right)$, at a given time *t* after an increase in concentration $C_{T}$, the asymptotic equilibrium value $S_{eq}$ and the saturation value $S_{\infty }$ are obtained by dividing $\sigma_{T}$, $\sigma_{eq}$ or $\sigma_{\infty }$ by the molecular mass of the ssDNA, respectively, where $\sigma_{\infty }$ and $S_{\infty }$ are the mass surface density and the surface number density of target at saturation reached at large $C_{T}$.

## 3. Results

### 3.1. Analysis of Binding Curves

**Figure 2:**
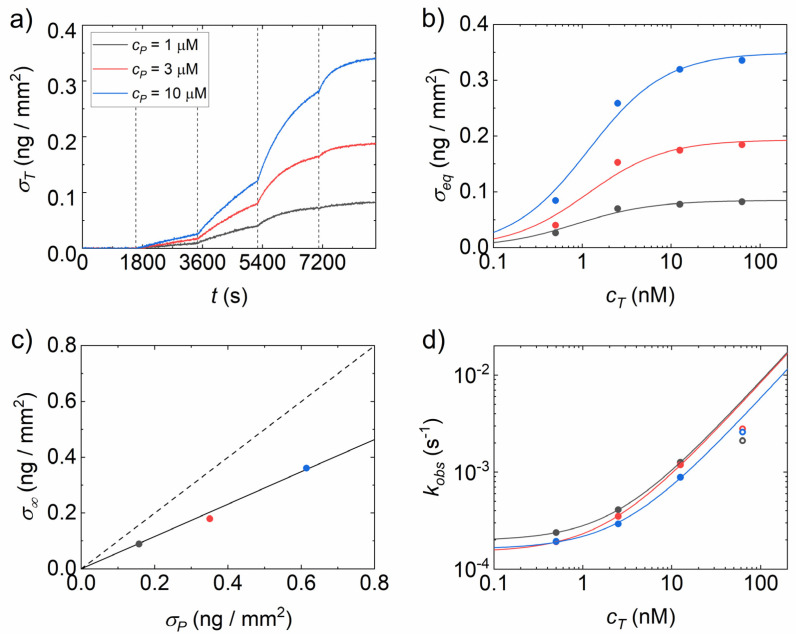
Analysis of RPI kinetic curves for DNA hybridization on MCP4 copolymer. (**a**) Binding curves for spots with three different DNA spotting concentrations: 1, 3 and 10 μM shown in grey, red and blue colour, respectively. Vertical dashed lines represent the additions of target DNA strand to concentrations of 0.5, 2.5, 12.5 and 62 nM. (**b**) Equilibrium asymptotic amplitudes obtained from exponential fits to binding curves in panel a. Lines represent fits with Equation (3). (**c**) Saturation values of target surface density as a function of probe surface density. The dashed line represents the hybridization of all probe strands, i.e., yield of 100%. The continuous black line is a linear fit, from which the yield was calculated to be ~60%. (**d**) Observed kinetic rates obtained from the exponential fits of the hybridization curves are reported in panel A. Lines represent linear fits with Equation (4). The data points at the largest concentration are excluded from the fit applying a procedure described in [13]. In panel b, c and d, the color indicates the spotting concentration as in panel a.

The hybridization of 23-*mer* targets in solution with fully complementary strands was measured by the RPI biosensing platform. The brightness of the ssDNA probe spots on the surface was converted into surface density of target molecules $\sigma_{T}$, as described in the Materials and Methods. The target strands were added in solution at increasing concentrations, and the kinetic binding curves were measured after each addition. Figure 2a shows the binding curves for an RPI sensing surface coated with MCP4 and spotted with different concentrations of probes, *C_P_*, in the spotting buffer. The amplitude of the binding curves increases with the target concentration in solution, as well as with *C_P_*. Figure 2b reports the equilibrium amplitudes $\sigma_{eq}$ of the individual binding curves of Figure 2a extracted from exponential growth fits (Equation (2)).

The concentration dependence of $\sigma_{eq}$ was fitted by a Langmuir adsorption model, according to Equation (3), from which the saturation amplitudes of captured strands $\sigma_{\infty }$ and the equilibrium constant for dissociation *K_d_* were obtained. $\sigma_{\infty }$ increases with *C_P_* as a consequence of the larger amount of surface-immobilized probe. The amount of probe in each spot was quantified in terms of surface density, $\sigma_{P}$, computed by Equation (1) and compared to the surface density of target $\sigma_{\infty }$. Figure 2c shows that $\sigma_{T}$ linearly scales with $\sigma_{P}$ and their ratio is 0.6, corresponding to 60% of surface probes accessible to target hybridization, in agreement with previous studies performed in similar experimental conditions but with shorter complementary strands [13]. Analogously, the values of *K_d_* extracted from the data in Figure 2b by fitting Equation (3) slightly increase with *C_P_*, as observed in previous works. This effect is primarily ascribed to an increased electrostatic repulsion due to the surface accumulation of DNA probes [28,29].

The analysis of the binding curves of Figure 2a also provides the kinetic parameters of the hybridization. The characteristic rate *k_obs_* extracted from the fit of each binding curve is reported in Figure 2d as a function of the target concentration in solution, together with linear fits according to Equation (4). The values of the kinetic rate for dissociation, *k_off_*, and association, *k_on_*, depend weakly on *C_P_*, and a larger set of data is required to extract a trend. At the largest concentration of target strands, at which the fraction of accessible probes hybridized by the targets is close to saturation, we observed bending of *k_obs_*(*C_T_*) ascribed to reduced access to the surface probes due to crowding, as reported in a previous study [13]. To further improve the analysis of the equilibrium and kinetic parameters and investigate the possible effect of the specific surface chemistry used for probe immobilization, the experiment shown in Figure 2 was repeated for different types of polymer coating.

### 3.2. Effect of Copolymer Coatings on Hybridization Yield

**Figure 3:**
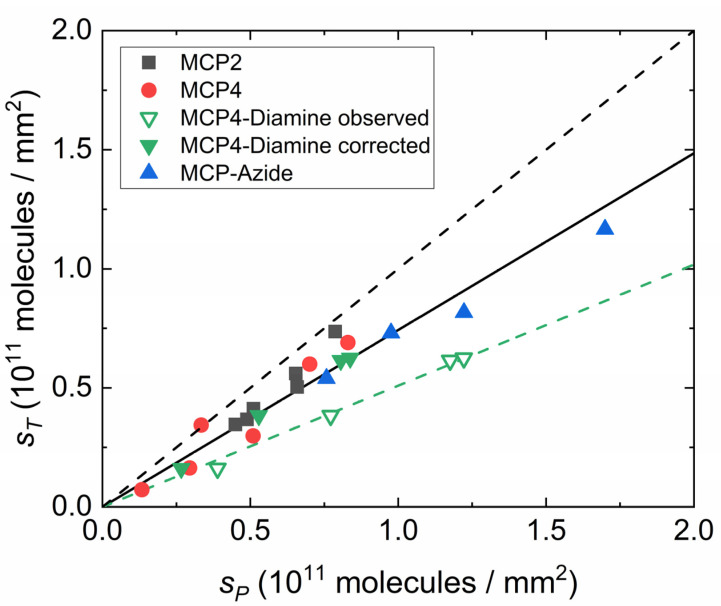
Hybridization yield for different copolymer coatings. The saturation amount of captured target strands per area is plotted as a function of the surface number density of the probe strands. The black continuous line is of a linear fit to the black, red and blue points. The green line displays a linear fit to the green, open points. The dashed line represents *S_T_* = *S_P_*, i.e., 100% yield.

We compared the hybridization of the 23-mer probe immobilized on different variants of MCP4 copolymer. We studied coatings with a slightly positive net charge (MCP4-Diamine), smaller density of sites for probe covalent immobilization (MCP2), or a different immobilization chemistry (MCP-Azide). The overall RPI signal provided the mass surface density of captured target $\sigma_{T}$, which is typically proportional to the mass surface density of immobilized probes $\sigma_{P}$. However, the scaling of $\sigma_{T}$ on $\sigma_{P}$ can depend on the details of the immobilization strategy. In order to investigate this dependence, we determined the amount of captured target on the different coatings at different spotting concentrations. The mass surface densities of probe and target obtained by RPI analysis were converted into the more general surface number densities, *S_P_* and *S_T_*, respectively, by dividing the mass by the corresponding molecular weight. As shown in Figure 3, the probe density spans one order of magnitude, and all the coatings displayed a linear relationship between *S_P_* and *S_T_*. The amount of captured target is generally lower than the total amount of probe on the surface because some of the probe strands are not available for hybridization, hence the overall yield is less than 100%. The linear dependence of *S_T_* on *S_P_* for each coating indicates a constant yield over the explored range. The data in Figure 3 indicate that the average hybridization yield on MCP4, MCP2 and MCP-Azide coating is around 75%, whereas the apparent yield of MCP4-Diamine (green, open triangles) is about 50%. However, since blocking with ethylenediamine occurs after the probe immobilization step, we ascribed the apparently larger values of *S_P_* for MCP4-Diamine to a slight swelling of the copolymer layer due to the positive charges, corresponding to about a 20% increase in thickness, which affects the coefficients of Equation (1). Therefore, we corrected the values of *S_P_* for this effect to recover the same range of values observed for the other copolymers (full, green triangles in Figure 3). Overall, the observed yield is slightly larger than what was previously observed for 12-*mer* hybridization on similar coatings, where yields of 50% ± 20% were reported [13].

### 3.3. Dependence of Association Rate on Probe Density

**Figure 4:**
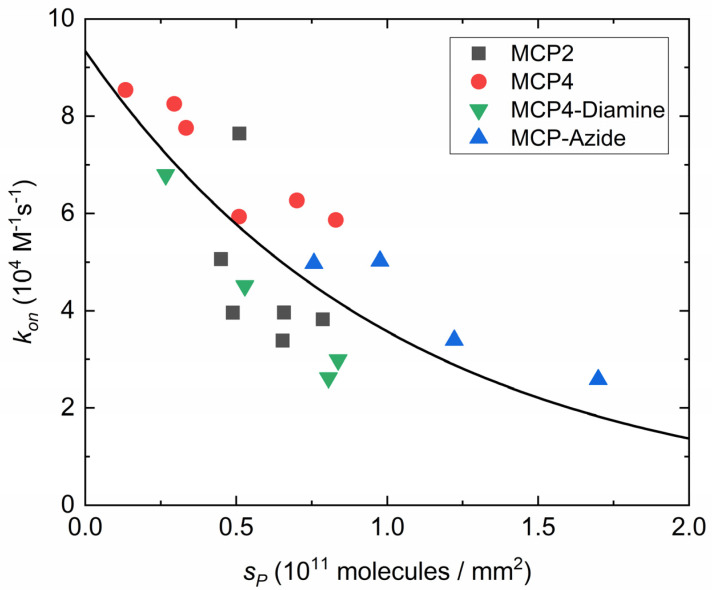
Association rate constant *k_on_* as a function of DNA probe density *S_P_*. The black curve represents an exponential fit to all data *k_on_*(*S_P_*) with Equation (5).

The distribution of probes on the surface is expected to primarily affect the association kinetics of DNA hybridization [13]. Therefore, we studied the dependence of *k_on_* on the surface density of probes *S_P_* for the four different copolymers. By varying the spotting concentration of probe strands, the different coatings were able to achieve slightly different ranges of *S_P_*. In particular, a probe surface density higher than 10^11^ molecules/mm^2^ was only obtained with MCP4-Diamine and MCP-Azide. From each dataset, the value of *k_on_* was extracted from the slope of *k_obs_*(*C_T_*), according to Equation (4). Figure 4 shows the behavior of *k_on_* as a function of *S_P_* for the different coatings. Following the kinetic analysis proposed in [13], the entire dataset of *k_on_*(*S_P_*) was fitted with a single exponential decay:

(5) $$k_{on}\left(S_{P}\right)=k_{on,0}e^{-2\gamma S_{P}}$$

where $k_{on,0}$ is the kinetic association rate in the absence of any effect inhibiting hybridization, such as electrostatic repulsion and steric effects (i.e., at low enough *S_P_*), and *γ* accounts for the repulsive effect per unit of probe density. The factor two at the exponent of Equation (5) approximates the term (1 + *nφ*) reported in [13] and accounts for the fact that the values of *k_on_* are extracted from Equation (4) for *C_T_* > *K_d_*. In the experiments reported here, the fraction of hybridized probes is *φ* ≈ 1, and the ratio *n* between probe and target length is 1.

Quite surprisingly, all investigated immobilization strategies are compatible with a common dependence of *k_on_*(*S_P_*) provided by Equation (5), hence confirming a predominant effect of electrostatic repulsion between nucleic acids over other possible effects mediated by the properties of the copolymer coatings. The fit of all values of *k_on_*(*S_P_*) with Equation (5) yields *k_on,_*_0_ = 9.34 ± 1.1 × 10^4^ M^−1^·s^−1^ and *γ* = 9.6 ± 2.1 × 10^−12^ mm^2^. The value *γ* is remarkably similar to that reported for 12-*mer* hybridization in [13], which is *γ* = 12 ± 1 × 10^−12^ mm^2^. In contrast, the measured value of *k_on,0_* for the 23-*mer* is about three times smaller than that reported for the 12-*mer*. A decrease in *k_on_* with the strand length was also observed by single-molecule experiments in solution [30]. Additional effects can contribute to decreasing the association rate on a surface, including the presence of DNA surface probes inaccessible for hybridization but contributing to the surface electrostatic potential [13] and transient weak interactions of the probes on the sensor surface [31].

### 3.4. Effect of Copolymer Coatings on the Equilibrium Constant for Dissociation

**Figure 5:**
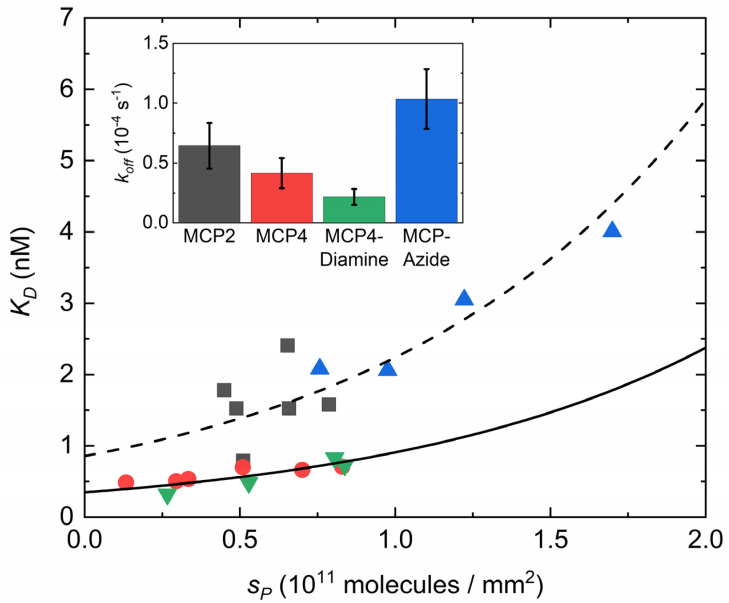
Equilibrium constant for dissociation $K_{d}$ The data points represent $K_{d}=k_{off}/k_{on}$ for DNA hybridization on different copolymers. The black continuous and dashed curves are fit with Equation (6) to red–green and black–blue points, respectively. Inset: average value and standard deviation for the dissociation rate constant $k_{off}$. The colors of points and curves of the main panel correspond to the copolymers as indicated in the inset.

The different values of the kinetic rate for association *k_on_* shown in Figure 4 affect the equilibrium constant for hybridization. In particular, the dissociation equilibrium constant *K_d_* = *k_off_*/*k_on_* tends to increase with *S_P_* because of the strong decrease in *k_on_* and a weak dependence of *k_off_* on *S_P_*. Indeed, the measured values of *k_off_* were independent on *S_P_* within the experimental uncertainties, although their averages differed for each type of surface coating. The inset in Figure 5 shows the observed average values of *k_off_*. The smallest values, i.e., the longest lifetimes for hybridization, were obtained for MCP4 and MCP4-Diamine coatings, whereas the MCP-Azide coating showed the largest *k_off_*. This behavior affects the value of the equilibrium constant *K_d_*. As reported in Figure 5, MCP2 and MCP-Azide coatings showed larger values of *K_d_* and a steeper dependence on *S_P_* than MCP4 and MCP4-Diamine. Assuming that all the dependence of *K_d_* on *S_P_* is accounted for by the behavior of *k_on_*(*S_P_*) given by Equation (5), the values of Figure 5 were fitted with an exponential dependence given by

(6) $$K_{d}\left(S_{P}\right)=K_{d,0}e^{2\gamma S_{P}}$$

where *K_d,_*_0_ = *k_off_*/*k_on,_*_0_ is the equilibrium dissociation constant at very small values of *S_P_* and *γ* is fixed to the value obtained by the fitting of *k_on_*(*S_P_*) shown in Figure 4. The good quality of fit for MCP4 variants supports the initial assumption.

## 4. Discussion

In most detection methods, including label-free biosensors and fluorescence microarrays, the limit of detection (LOD) scales generally with the maximum surface density of target strands that can be captured by the sensing surface. In contrast, both kinetics and equilibrium parameters are typically degraded at a large surface density of immobilized probe strands [8,32]. Therefore, depending on the objectives of the analytical method, an optimal surface density of both immobilized probes and captured targets at saturation should be sought. These two quantities are related through a linear scaling, at least for probe and target surface density lower than the maximum packing limit. Assuming a lateral size of double-strand DNA of 2 nm and a maximum random surface packing in 2D of 80%, the steric limit is about 2.5 × 10^11^ molecules mm^−2^. Remarkably, as shown in Figure 3, the linear dependence between *S_P_* and *S_T_* was confirmed up to 1.7 × 10^11^ molecules mm^−2^, hence it is close to the theoretical packing limit. Despite this, as shown in Figure 4, the effect of probe surface density on the association kinetics due to electrostatic repulsion is relevant down to about 10^10^ molecules mm^−2^. In agreement with previous studies [13,33], this behavior is found to be common to all coatings considered.

Differences due to the copolymer coatings arise when considering the hybridization yield and the equilibrium constant. The MCP-Azide copolymer leads to the largest values of *S_P_*, and hence of *S_T_* (Figure 3). Therefore, MCP-Azide coating provided the largest asymptotic amplitude of hybridization signal. This feature is particularly relevant for endpoint methods such as fluorescence microarray. However, MCP-Azide copolymers also showed a rather large equilibrium constant *K_d_* (Figure 5), which implies smaller signals at target concentrations around *K_d_* or lower. This value of *K_d_* originates from large values of *k_off_*, which are possibly due to suboptimal distribution of bonding sites for DNA probe covalent immobilization or to spurious interactions involving the aromatic DBCO moiety. Previous studies have shown that amine/NHS and azide/alkyne reactions for DNA immobilization on DMA-based copolymers provide similar signal amplitudes in fluorescence microarrays [15,20]. This is consistent with the combination of two competing properties observed here: a larger probe density, but also a larger *K_d_* of MCP-Azide.

At the other extreme, MCP4 and MCP4-Diamine coatings displayed a lower range of amplitudes (Figure 3), and smaller *K_d_*, even after correcting the probe surface density (Figure 5). Relative to MCP4, MCP4-Diamine, which bears additional positive charges, showed slightly slower dissociation kinetics, but also smaller *k_on_*. We interpret this effect being due to a weak attractive electrostatic interaction of the positively charged copolymer with both probe and target DNA. Considering the target strands, this interaction can reduce the dissociation rate or favor rebinding. In contrast, the same copolymer–DNA interaction applied to probe strands can compete with hybridization and reduce the association rate. The two effects compensate, hence yielding similar values of *K_d_* for MCP4 and MCP4-Diamine.

MCP4 and MCP2 coatings provided hybridization yields similar to or larger than the MCP-Azide coating, but in contrast to this polymer they did not allow for probe surface densities larger than 10^11^ molecules mm^−2^ in the spotting conditions explored here. Despite the similar asymptotic signal amplitude, MCP4 and MCP2 coatings differ in terms of *K_d_*. Surprisingly, the reduced density of covalent sites provided by MCP2 is associated with larger values of *K_d_*, due to both slightly smaller *k_on_* and larger *k_off_*. We speculate that this effect can be ascribed to the reduced variety of the positions of the immobilization sites on the copolymer during the spotting. In contrast, on the MCP4 coating, the DNA probes can better adapt to the surface features given by copolymer conformation and DNA neighbors because of the larger number of sites for covalent binding. These results are consistent with the fact that the surface density of active esters in MCP4 copolymer was estimated at about 10^12^ sites mm^−2^ [18], hence at about an order of magnitude larger than the surface density of immobilized DNA probes. The comparison between MCP4 and MCP2 hybridization parameters suggests that such large amounts of immobilization sites are critical to enable an optimal distribution of probe strands on the copolymer.

## 5. Conclusions

Surface-based methods to measure biomolecular interactions, including label-free biosensors and fluorescence microarrays, rely on a proper immobilization of probes. The physical–chemical properties of the surface environment can affect the interaction between the probes and the targets in solution. In the case of protein–protein interaction, e.g., antibody–antigen, in which the binding occurs via a localized recognition site of large-structured molecules, surface properties mainly affects signal amplitudes, but have small effects on the interaction parameters [34]. In contrast, the details of surface immobilization strongly affect nucleic acids hybridization, in which the binding involves the conformational locking of flexible chains, and even weak spurious interactions with a single monomer can reduce the hybridization affinity [31]. On one side, the sensitivity of hybridization to surface immobilization makes it difficult to reach the rapid kinetics and the large affinities observed for freely diffusing strands in solution. On the other, NA probe surface immobilization can offer additional control parameters to optimize the assay [13].

Equilibrium and kinetics parameters for DNA hybridization on a surface have been experimentally measured in previous works by several techniques and in different conditions. Table 1 reports selected examples of some of these studies in comparison with the results of this work. Although the values of the extracted parameters are generally in agreement among different studies, large discrepancies are observed depending on the detailed experimental conditions. In this work we addressed the effect of type and surface density of sites for covalent immobilization of DNA probes on a nanoscale hydrogel coating, as well as different charges of the copolymer backbone. It is confirmed that the surface density of DNA probes *S_P_* is the main parameter affecting the hybridization behavior. Therefore, the comparison between different surface coatings requires the analysis of hybridization as a function of *S_P_*, which is not trivially related to the surface density of covalent immobilization sites for the probes on the copolymer. As reported in Table 1, in comparison to previous studies, we examined a wide range of *S_P_* values. This enabled us to apply the analysis based on Equations (5) and (6) in order to rule out the dominant effect due to surface probe density.

According to the results of this work, different immobilization strategies can be preferred depending on the application. We found that azide/alkyne immobilization enables the achievement of larger values of *S_P_* relative to amine-based immobilization maintaining large hybridization yields. Therefore, this strategy is preferred when the amplitude of the signal is the main optimization parameter of the assay. In contrast, the lowest values of equilibrium dissociation constants were obtained with MCP4 and MCP4-Diamine coatings, which provide a large density of amine-based covalent sites. Accordingly, these types of coatings are preferred when sensitivity to small concentrations of target strand is the main goal of the assay design.

Based on the results of this work, quantitative criteria can be applied to select optimal surface-immobilization coatings for DNA microarrays depending on the application, either based on fluorescence or label-free detection. Moreover, this work provides a reference framework on methodology for further studies on the effect of DNA immobilization strategies and their characterization.

## Figures and Tables

**Table 1:** Comparative table reporting examples of surface DNA hybridization experiments.

Reference	Measuring Method	DNA Length (# Bases)	Surface Coating	DNA Probe Surface Density (10^10^ Molecules mm^−2^)	*K_d_* (nM)	*k_on_* (10^4^ M^−1^ s^−1^)	*k_off_* (10^−4^·s^−1^)
Jensen et al., 1997 [35]	Surface plasmon resonance	15	Dextran and streptavidin		25	1.2	2.9
Nelson et al., 2001 [36]	Surface plasmon resonance	18	11-mercaptoundecyl amine	1	55		
Peterson et al., 2002 [37]	Surface plasmon resonance	25	-	1.5–3	16		
Gao et al., 2006 [38]	Surface plasmon resonance	25	-	4.5–6.8		5.7	
Irving et al., 2010 [39]	cyclic voltammetry	18	-	5	770		
Ozkumur et al., 2010 [16]	Spectral reflectance imaging biosensor	20	MCP4				4.1
Qiao et al., 2015 [9]	Total internal reflection fluorescence	25	Aldehyde	2.9	10^−7^–2 ^a^		
Nava et al., 2016 [31]	RPI (perfluoropolymer substrate)	12	MCP4	2–9	1–1.8	2–10	6–10
Sola et al., 2019 [15]	Interferometric reflectance imaging sensor	23	MCP4	7.8			
			MCP-Azide	11.6			
Vanjur et al., 2020 [13]	RPI	12	MCP4–MCP2	3–9	1–11	6–40	1.4–9
*This work*	RPI	23	MCP4	1.3–8.3	0.5–0.7	5.9–8.5	0.4
			MCP4-Diamine	2.6–8.3	0.3–0.8	2.6–6.9	0.2
			MCP2	4.5–7.9	0.8–2.4	3.4–7.6	0.6
			MCP-Azide	7.5–17	2–4	2.6–5	1

^a^ Estimated from melting measurements.

## Data Availability

Data are contained within the article.
